# A Pilot Single-Site Randomized Control Trial: Investigating the Use of Donor Milk in the Late Preterm and Term Infant in the Neonatal Intensive Care Unit

**DOI:** 10.21203/rs.3.rs-2540272/v1

**Published:** 2023-02-08

**Authors:** Neema Pithia, Tristan Grogan, Meena Garg, Kalpashri Kesavan, Kara Calkins

**Affiliations:** UCLA, Los Angles, California; UCLA, Los Angles, California; UCLA, Los Angles, California; University of California; University of California

## Abstract

**Objective:**

We aimed to study the use of donor milk (DM) in term and late preterm infants (LPIs) when mother’s own milk (MOM) was unavailable. We hypothesized this study would be feasible and breastfeeding attempts and the percentage of MOM (MOM%) would increase with DM without adversely affecting growth.

**Study Design:**

This was a pilot study (n=32). Infants with gestational age >34 weeks admitted to the neonatal intensive care unit were included. Infants were randomized to: the human milk (MOM+DM) or formula (MOM+F) groups.

**Result:**

Consent rate was 52%. Breastfeeding attempts increased significantly over time in the MOM+DM group compared to the MOM+F group (group p=0.41, time p =0.02, group*time p=0.01). Growth at multiple time points was similar when the two groups were compared.

**Conclusion:**

A study randomizing term infants and LPIs to DM or formula when MOM is unavailable is feasible. DM may increase breastfeeding attempts without compromising growth.

## Introduction

For most newborn infants, breast milk is best source of nutrition. Breast milk is associated with a decrease in the risk of infection and childhood disorders.^1^ For mothers, breastfeeding has numerous benefits. Like infants, it can reduce the risk of metabolic syndrome as well as decrease the incidence of certain cancers.^1^ Despite the known benefits, rates for initiating and sustaining breast milk production are lower for mothers with infants in the neonatal intensive care unit (NICU) than mothers with infants in the well-baby nursery.^1,2^ Donor milk supplementation may help overcome breastfeeding barriers and improve breastfeeding rates for mothers and infants in the NICU.

For very low birth weight infants VLBWs (birth weight < 1500g), donor milk (DM) is the preferred alternative when mother’s own milk (MOM) is unavailable.^3,4^ In VLBWs, DM supplementation, when part of an all human milk diet, is associated with a decreased incidence of necrotizing enterocolitis (NEC), late-onset sepsis, retinopathy of prematurity, chronic lung disease, increased breastfeeding rates at hospital discharge, and improved neurodevelopment.^4^ For these reasons, the American Academy of Pediatrics recommends using DM for VLBWs when MOM is unavailable.^5^ Likewise, the World Health Organization and United Nations Children’s Fund (UNICEF) recommend DM supplementation for all infants, regardless of their birth weight or gestational age.^6^ Despite these studies and recommendations, it is not standard practice to provide DM supplementation to term and late preterm infants (LPIs) who are hospitalized in the NICU.^2,4^

Regardless of the potential benefits of an all-human milk diet, some people have raised concerns about DM. First, unfortified DM provides less energy, protein, minerals, and electrolytes than standard formula and MOM.^2^ Second, DM is pasteurized to minimize infection risk. This process destroys harmful and beneficial microbiota. Pasteurization decreases the number of immunoglobulins, protective cytokines, growth factors, macronutrients, and hormones. Lastly, DM increases the costs of a NICU stay depending on the volume of MOM provided. A retrospective analysis of 319 VLBW infants showed that the median price (in US dollars) of DM in 2016 was $21.18 per 100mL whereas the median price of formula was $3.30 per 100mL.^7^

Data on DM use in the late preterm infant (LPI) and term infant in the NICU is scant.^8^ Hence, this pilot study aimed to determine the feasibility of a study that randomized LPIs and term infants in the NICU to DM or formula supplementation when MOM was unavailable. The study’s secondary outcomes were breastfeeding attempts and the percentage of MOM (MOM%) consumed over a 48-hour period at the end of seven days of supplementation or at NICU discharge, whichever occurred sooner, and growth at the end of the study intervention, NICU discharge, and 6–8 weeks chronological age. We hypothesized that this study would be feasible, and when compared to formula supplementation, DM supplementation would be associated with an increase in breastfeeding attempts and MOM% without adversely affecting growth.

## Methods

This study was conducted at two NICUs at the University of California, Los Angeles (Mattel Children’s Hospital at Ronald Regan Hospital and Santa Monica Medical Center). This study was approved by the local institutional review board and registered at clinicaltrials.gov (NCT04572581). All parents and legal guardians provided written informed consent for themselves and their infants.

This study’s primary outcome was feasibility, defined by rates for consent, study completion, and study diet adherence. Secondary outcomes included breastfeeding attempts and the MOM% consumed over 48 hours prior to NICU discharge or at the end of the seven day study intervention, whichever came sooner, along with growth (weight, length, and head circumference) at the end of the study intervention, NICU discharge, and 6–8 weeks chronological age.

### Participants

Inclusion criteria included infants with a gestational age > 34 weeks, < 48 hours of age, NICU admission, predicted NICU stay >72 hours, and a mother who intended to breastfeed. Exclusion criteria included genetic syndromes that affect growth or feeding, mechanical ventilation, common breastfeeding contraindications, major congenital anomalies, or any infant whose care was considered futile.

### Sample size

We aimed to approach 64 participants and presumed a consent rate of 50%. With 16 patients per group, we were adequately powered (> 80%) to detect standardized effect sizes as small as 1.03 between groups (two sample t-test, alpha = 0.05, two-tailed) for continuous variables and differences between groups of 48% (e.g., 26% vs. 74%) using a chi-square test (alpha = 0.05, two-tailed) for categorical variables.

### Randomization and Intervention

Eligible participants were approached in the mother’s hospital room 48 hours before or after delivery. Sealed envelopes were used for randomization. Twins were randomized individually. Infants were randomized to one of two groups. If the infant was randomized to the human milk group (MOM + DM group), the infant received DM when MOM was not available. If the infant was randomized to the formula group (MOM + F group), the infant received formula when MOM was not available.

This was not a blinded study; the parents/legal guardians and primary medical team were aware of the assigned diet. The primary medical team determined the formula type, feeding volume and route, fortification, and need for parenteral nutrition. The study intervention ended after seven days of life or if the subject was transferred to the well-baby nursery or discharged from the NICU prior to seven days of life.

### Data Collection

Demographic information was collected on each mother-infant dyad. Race, ethnicity, education, and income were determined using the United States Census Bureau 2020 standard categories.^9^ Data on growth, type and amount of milk intake, and fortification were collected. Growth was assessed using Z-scores.^10^

### Follow-up

At 6–8 weeks chronological age, data on growth were collected via the electronic medical record or conversations with the Pediatrician. The mother completed a survey via telephone or email to assess feeding practices and breastfeeding goals and barriers.

### Statistical Analysis

Patient characteristics and study variables were summarized by group (MOM + DM, MOM + F) using means/SD, medians (IQR) or percentage (frequency) unless otherwise noted. Groups were compared using the chi-square test for categorical variables and the student t test or wilcoxon rank test for normal and non-normal continuous variables, respectively. Tests were performed using GraphPad Prism version 8.0.0 (GraphPad Software, San Diego, California USA, www.graphpad.com.

For assessing study outcomes measured over time (MOM%, number of breastfeeding attempts, growth), linear mixed-effects models with terms for group (MOM + DM, MOM + F), time, and an interaction variable (group*time with a random patient effect) were performed. Due to the higher number of twins in the MOM + F group, a post-hoc analysis was performed in the same manner examining the mother’s total attempts at breastfeeding, regardless of the number of infants, and examining the average MOM% provided over specific time periods if the mother had two infants in the study. These analyses were run using SAS V9.4 (Cary, NC), and p-values < 0.05 were considered statistically significant.

## Results

Between December 2020 and February 2022, 62 parents were approached, and 32 LPI and term infant and parent dyads were enrolled. The study’s consent rate was 52%; 100% of participants completed the study; diet adherence was 88% and 100% for the MOM + DM group and MOM + F group, respectively. Diet deterrence in the MOM + DM group consisted of one formula feed in one subject (Fig. 1).

The groups had similar parental characteristics. The majority of parents self-identified as White, non-Hispanic/non-Latino. The median age (IQR) of the mother was 33 years (31–37 years) and 33 years (30–35 years) in the MOM + DM group and MOM + F group, respectively (p = 0.7). The mean (SD) gestational age in both groups was 35±1 weeks, and birth anthropometrics were comparable. The number of twins was higher in the MOM + F group compared to the MOM + DM group. There were four sets of twins from the same mother in the MOM + F group; and one in the MOM + DM group (50% vs. 6% p = 0.01) (Table 1).

**Figure 2:**
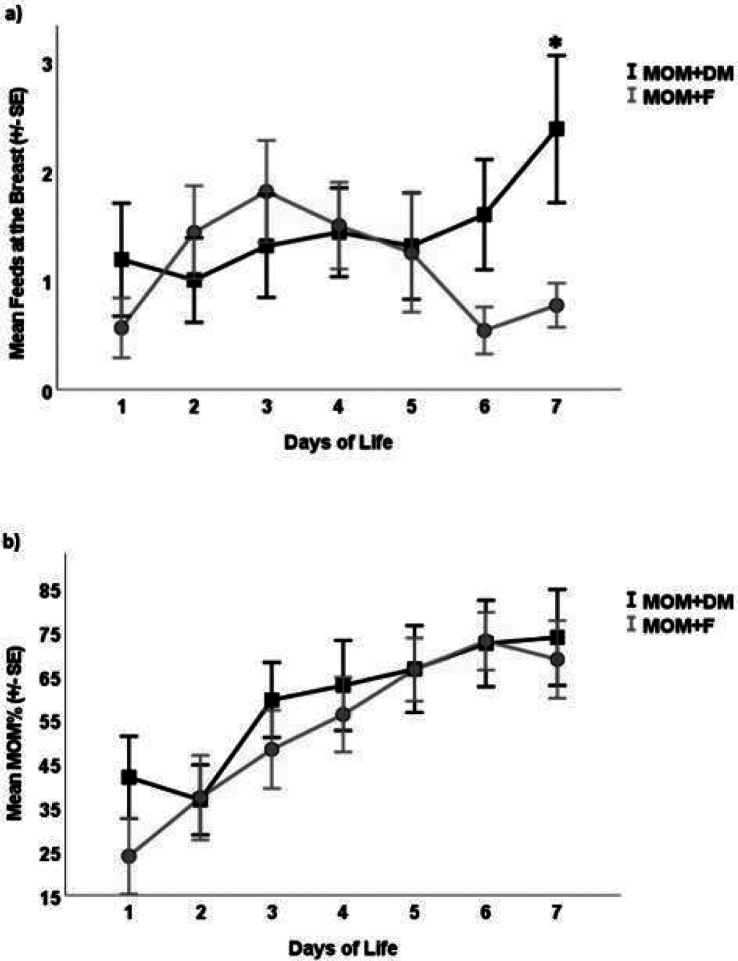
Number of feeds at breast and percentage of mother’s own milk consumed over time. n=16. MOM; mother’s own milk. DM; donor milk. F; formula. **a)** Number of feeds at breast for each day of life in the MOM+DM and MOM+F groups. Error bars represent one standard error. P-values from mixed effects models are: Group p=0.41, Time p=0.02, Group*time p=0.01, *p=0.03 using Student’s t-test. **b)** Percentage of MOM as a percent of the total feeds for each day of life. Group p=0.23, Time p<0.001, Group*time p=0.16.

Over time, the number of attempted feeds at the breast by the infant increased at a higher rate in the MOM + DM group compared to the MOM + F group (group p = 0.41, time p = 0.02, group*time p = 0.01) (Fig. 2a). However, the MOM% increased at a similar rate in both groups (group p = 0.18, time p = 0.05, group*time p = 0.16) (Fig. 2b). The post-hoc analysis revealed similar results for the number of attempted feeds at the breast (group p = 0.18, time p = 0.05, group*time p = 0.02) and the MOM% (group p = 0.53, time p < 0.001, group*time p = 0.38).

Infants in the MOM + DM group demonstrated a trend towards more breastfeeding attempts 48 hours prior to NICU discharge or at the end of the study intervention, whichever came sooner, when compared to the MOM + F group (median (IQR) 3.5 (1.5–6) vs. 1.5 (0.5–4) times, p = 0.1). However, this was not statistically significant. The MOM% consumed by the infant over the aforementioned time period did not differ (60% in the MOM + DM vs. 59% in the MOM + F group, p = 0.9).

**Figure 3:**
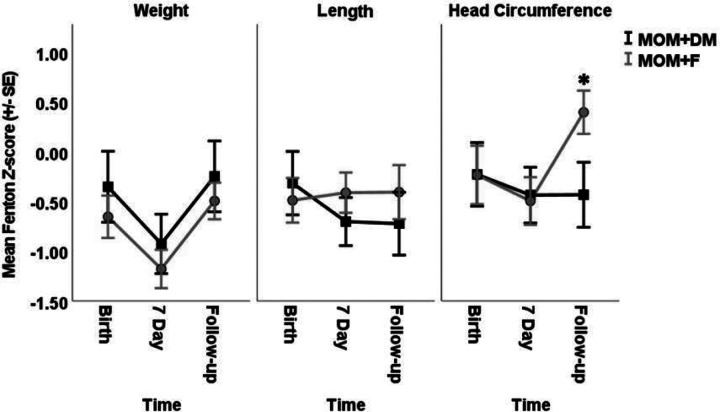
Weight, Length, and Head Circumference (HC) measured at birth, 7 days of life, and follow-up (6–8 weeks of age) for each group. n=16. MOM; mother’s own milk. DM; donor milk. F; formula. Error bars represent one standard error. Z-scores were obtained using PediTools Fenton 2013 data. P-values from the mixed effects models were: Weight; Group p=0.47, Time p=0.99, Group*time p=0.84. Length; Group p=0.81, Time p=0.16, Group*time p=0.09. Head Circumference; Group p=0.63, Time p=0.42, Group*time p=0.04. *p=0.04 using Student’s t test.

Mean (±SD) weight, length, and head circumference z-scores at the end of the study intervention (−0.9±1.2 vs. −1.2±0.8, p = 0.5; −0.7±1.0 vs. −0.4±0. p = 0.4, −0.4±1.1 vs. −0.4±1.0, p = 0.9, respectively) and NICU discharge (−1.0±1.2 vs. −1.2±0.9, p = 0.3; (−0.9±1.3 vs. −0.5±0.9, p = 0.2; (−0.5±1.1 vs. −0.3±0.8, p = 0.2) were not significantly different in the MOM + DM group and MOM + F group, respectively. Likewise, at follow-up, mean z-scores for weight and length for the MOM + DM group and MOM + F group were comparable. However, the MOM + F group had a significantly higher head circumference z-score compared to the MOM + DM group at 6–8 weeks chronological age (−0.4±1.3 vs. 0.4±0.9, p = 0.04) (Fig. 3).

Infants in both groups were prescribed fortified formula at discharge, 75% in the MOM + DM group and 88% in the MOM + F group received a fortified formula (p = 0.6). At 6–8 weeks chronological age, 25% and 13% of the infants were receiving exclusive formula, 44% and 50% received a mixed diet, and 31 % and 38% received exclusive breastmilk (p = 0.7 for all) in the MOM + DM group and MOM + F group, respectively. Barriers to breastfeeding are described in Table 2.

## Discussion

This pilot randomized controlled study demonstrated feasibility. While the study’s consent rate was 52%, there was high compliance with the dietary intervention. In a systematic review of ten randomized controlled trials, the median percentage consented (IQR) for neonatal clinical trials with a nutritional intervention in high-income countries was 79% (63–93).^11^ In our opinion, conducting this study during the Covid-19 pandemic presented unique and unexpected barriers to the informed consent process. Consent rates may be higher in the post-Covid-19 era.

When compared to MOM, DM has a lower nutritional content.^2^ In this study, growth was similar when the two groups were compared at the end of the study intervention, NICU discharge, and follow-up. The mean head circumference was significantly greater at 6–8 weeks in the MOM + F group compared to the MOM + DM group. The higher average head circumference was likely due to two outliers and a small sample size. Head circumference measurements are prone to error due to molding at birth and inconsistent measurement practices.

In the past, VLBW studies documented poor growth with DM when compared to both preterm formula and MOM.^12,13^ DM fortification and feeding protocols have helped mitigate this discrepancy.^14,15^ LPIs and term infants are thought to be less vulnerable to postnatal growth failure compared to VLBW infants.^16^ This study excluded critically ill infants who required mechanical ventilation, who may be more susceptible to postnatal growth failure than non-mechanically ventilated infants.^17^ It remains unclear if the inclusion of more chronically ill patients and the use of unfortified formula at discharge would have altered the subjects’ longitudinal growth.

The infant diets at follow-up were not significantly different between the two groups. The majority of women in both groups cited that they were providing formula to their infant at the time of follow-up because their doctors advised them to. The mean gestational age of infants in both groups was 35 weeks which would be considered a LPI. A systematic review done by Young *et al*. found that growth during the first six months in preterm infants (< 35 weeks) did not differ between those receiving preterm or standard term formula at discharge.^18^ The authors of this study concluded that “current recommendations to prescribe ‘postdischarge formula’ for preterm infants after hospital discharge are not supported by available evidence.”^18^ Of note, the majority of patients in this study were VLBW infants but some LPIs were included. Regardless of the lack of evidence, majority of infants in both groups were prescribed fortified formula at discharge. In addition to heeding their physician’s advice, some women were concerned about their infant not gaining enough weight and many women cited no specific reason for giving formula. These findings highlight the strong provider influence on breastfeeding and formula usage, which is vulnerable to implicit and explicit bias.

Additional secondary outcomes included breastfeeding attempts and MOM% 48 hours prior to NICU discharge or at the end of the study intervention, whichever was sooner. These outcomes were not significantly different between the two groups. However, the MOM + DM group had more breastfeeding attempts over time compared to the MOM + F group. A retrospective study of the Italian Neonatal Network of 4277 VLBW infants demonstrated an increase in exclusive breastfeeding at discharge when DM was available during hospitalization.^17^

The MOM + F group had more twins than the MOM + DM group. The twin burden may have limited the number of times mothers in the MOM + F group were able to put each infant to breast. However, our post-hoc analysis confirmed our results. A larger study with equal representation of twins in each cohort is required to validate this study’s outcomes. Alternatively, a twin-only or twin-excluded iteration of this trial may identify clinically relevant differences.^19^ Twins often have lower breastfeeding rates than singletons.^20,21^ An observational study of 26,000 twins and singleton VLBW infants showed that, on multivariate analysis, twin pregnancy had a statistically significant, albeit minimal, effect on breastfeeding cessation at NICU discharge (OR 1.10; 95% CI: 1.02,1.19).^22^ Although the number of times the infant was put to breast was higher in the MOM + DM group compared to the MOM + F group, the percentage of MOM 48 hours prior to NICU discharge remained similar in both groups. We can conclude that mothers in the MOM + F group were able to maintain their MOM supply for both twins. The ability of mothers to sustain their milk supply is complex and challenging and cannot be attributed to one factor alone.^23^

A similar number of women in both groups cited that they were not exclusively breastfeeding at follow-up due to concerns for an inadequate milk supply. There may be many reasons for this result. First, many mothers of infants admitted to the NICU have pregnancy-related conditions. Mothers with pregnancy induced hypertension, gestational diabetes mellitus, and obesity have a higher risk of delayed lactation and early cessation of breastfeeding.^24–26^ Second, the inability of the mother and infant dyad to room-in in the NICU may contribute to decreased MOM supply.^27^ A mother’s separation from her infant requires her to pump milk, which requires supplies and time. If the mother does not have access to a pump, either through her own financial means or the hospital, this presents a barrier. The majority of couples in this study were at least four times above the national poverty line and had a college or graduate degree. This may have increased the ability of mothers in both cohorts to obtain a pump and breastfeed. Low breastfeeding rates are associated with public insurance, transportation issues, lack of lactation support, preconceived cultural notions of the superiority of formula, and unaccommodated language barriers.^28–30^ Future studies should consider these barriers in their research.

There are racial disparities in DM provisions and breastfeeding rates in the US.^31^ The majority of mothers in this study were White. Fourteen and nine percent of the population is Black in the United States (US) and Los Angeles County, respectively.^9^ The number of Hispanic mothers was similar in both groups with 31 % in the MOM + DM group and 25% in the MOM + F. Nineteen percent and 49% of the population is Hispanic in the US and Los Angeles County, respectively.^9^ While the population of this study is more reflective of Los Angeles than the national population, it lacks socioeconomic and racial diversity, which may limit its generalizability.

Admission to the NICU is inherently fraught with caregiver stress affecting decisions to initiate and continue breastfeeding.^32^ Some mothers may feel guilty and blame themselves for complications that may have led to a NICU admission.^33^ This guilt compounded with stress and anxiety may lead to delayed and decreased lactogenesis. Milk production for both groups followed a similar trajectory reaching a plateau at postpartum day five. Most women are expected to reach an adequate supply between postpartum days three and five. We opted to provide the study intervention for seven days for this reason. On the contrary, a prospective study of 126 mothers suggested that it can take up to two weeks until 440mL of milk is available, which is the accepted lower limit of normal for established milk production.^34^ It is unclear if providing DM for more than 7 days would have changed the results of this study.

The small sample size limits this study’s generalizability. Ideally, this study should have been blinded to help eliminate bias. Studies on the economic benefit of DM are warranted given the cost of DM. Long-term follow up is also required to determine if an all-human milk diet in LPIs and term infants improves neurodevelopmental outcomes and decreases the incidence of childhood disorders and diseases, such as atopy, infections, and diabetes mellitus.

In conclusion, a study randomizing term infants and LPIs to DM or formula when MOM is unavailable is feasible. DM can potentially increase feeding attempts at the breast which may translate to improved breastfeeding longevity and fewer perceived barriers to breastfeeding for mothers. DM did not negatively affect growth and may be a viable option for supplementation when MOM is not available. Large-scale studies are needed that utilize both quantitative and qualitative methods to validate this study’s results.

## Figures and Tables

**Fiaure 1:**
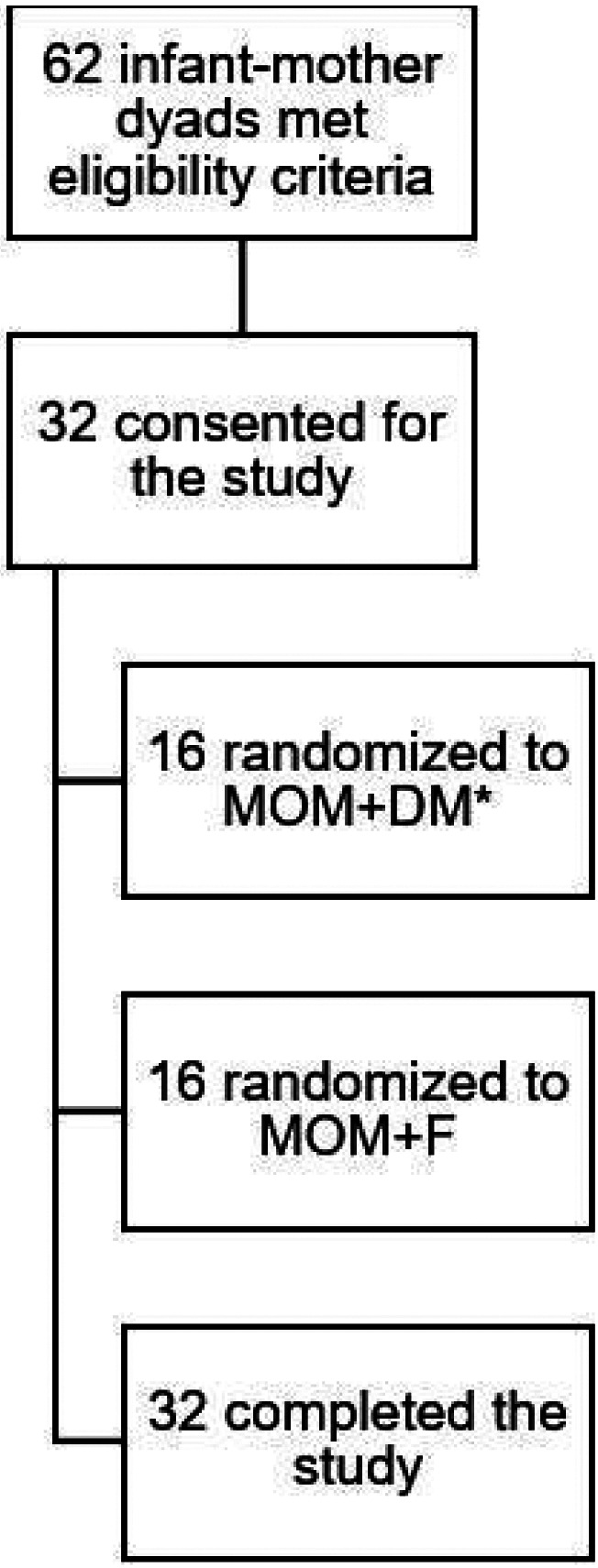
Study consent and completion rate. MOM; mother’s own milk. DM; donor milk. F; formula. *Infant fed with formula for one feed in MOM+DM group was not excluded from the study given the limited deviation.

**Table 1.** Characteristics of the Study Population.

	MOM+ DM (n=18)	MOM+F (n=18)	*P* value
**Maternal Characteristics**			
Race			
While	8 (50)	8 (50)	0.3
Asian	3 (18)	5 (31)	
Black or African American	0	1 (G)	
Native Hawaiian or Other Pacific Islander	1 (6)	0	
More than One Race	2 (13)	2 (13)	
Unknown/Not Reported	2 (13)	0	
Hispanic or Latino	5 (31)	4 (25)	0.7
Age (years)	33±4	33±3	0.7
Highest Level of Education			
Less than High School	0	0	0.2
High School Graduate	2 (13)	5 (31)	
Some College	1 (6)	2 (13)	
Collage Graduate	6 (38)	5 (31)	
Graduate Degree	7 (44)	7 (44)	
Gross Annual income			
Up to $9,875	0	3 (19)	0.3
$9,876–$40,125	5 (31)	2 (13)	
$40,125–$95,526	3 (19)	3 (19)	
$85,525–$163,300	5 (31)	7 (44)	
$163,300–&270,350	2 (13)	0	
$207,350–$518,400	1 (6)	1 (6)	
More than $518,400	0	0	
Pro-pregnancy EMI (kg/m^2^)	23±4.7	22.4±2.9	0.B
Para Status	0.6±0.6	0.5±0.7	0.B
Charioamnioritis	1 (6)	0 (0)	0.3
Gestational Diabetes	2 (13)	2 (13)	1.0
Pregnancy-Induced Hypertension	7 (44)	4 (25)	0.7
**Infant Characteristics**			
Geslational Age (weeks)	4311	35±1	0.4
Fomale	5 (31)	8 (50)	0.7
Vaginal Delivery	6 (38)	5 (31)	0.7
Fetal Growth Restriction	5 (31)	7 (44)	0.5
Twins	1 (6)	8 (50)	0.01
Birth Weight (g)	2332±599	2129±457	0.3
APGAR – 5 min	8.6±0.6	8.5±0.5	0.5
Admission Diagosis			0.3
Prematurlty	4 (25)	6 (38)	
Respiratory	12 (75)	10 (63)	
Non-invasive Respiratory Support	14 (88)	10 (63)	0.1
Length of NICU Stay (days)	15±8	14±8	0.6
Day or First Feed	0.6±0.8	0.8±0.6	0.6
NPO days	2±1.9	1.6±0.9	0.5
Parenteral Nutrition Days	4.2±3.6	2.8±1.7	0.2
Type of Nutrition at First Feed			
MOM	5 (31)	2 (13)	0.4
DM	2 (13)	n/a	
MOM+DM	14 (88)	n/a	
MOM+F	n/a	7 (44)	
F	n/a	4 (25)	
Fortified Formula Prescribed at Discharge	12 (75)	14 (88)	0.6

M; mother. MOM; mother’s own milk. DM; donor milk. F; formula, BMI; body mass index, NPO; nil per os. Data are represented as means±SD and n (%). Analysis by Student’s t-test and ANOVA. Race and Ethnicity, Highest Level of Education, and Gross Annual Income were self-identified by study participants.

**Table 2:** Reasons for Using Formula.

	MOM+DM (n = 16)	MOM + F (n = 16)	*P* value
I wanted to provide formula	2 (13)	2 (13)	1.0
I did not have enough breastmilk	5 (31)	6 (38)	1.0
My baby could not latch	2 (13)	0	0.5
My doctor told me to	8 (50)	8 (50)	1.0
I did not think my breast milk provided enough nutrition	1 (6)	2 (13)	1.0
My baby was losing too much weight	2 (13)	1 (6)	1.0
My baby was jaundice	0	0	1.0
I was afraid my baby would not gain weight with breast milk	0	3 (19)	0.2
There is no place for me to pump at work	1 (6)	0	1.0
Other	1 (6)	0	1.0
No reasons identified	5 (31)	6 (38)	1.0

MOM; mother’s own milk. DM; donor milk. F; formula. Survey administered to mothers via phone or email when infant was 6–8 weeks chronological age. Mothers were able to choose multiple reasons for using formula. Data represented as n (%). Analysis by Fisher’s exact test.
